# The bacterial parasite *Pasteuria ramosa* is not killed if it fails to infect: implications for coevolution

**DOI:** 10.1002/ece3.438

**Published:** 2012-12-19

**Authors:** Kayla C King, Stuart K J R Auld, Philip J Wilson, Janna James, Tom J Little

**Affiliations:** 1 Institute of Integrative Biology, University of LiverpoolCrown Street, Liverpool, L69 7ZB, UK; 2 Institute of Evolutionary Biology, School of Biological Sciences, University of EdinburghAshworth Labs, King Buildings, Mayfield Road, Edinburgh, EH9 3JT, UK

**Keywords:** *Daphnia*, dilution effect, host–parasite coevolution, *Pasteuria*

## Abstract

Strong selection on parasites, as well as on hosts, is crucial for fueling coevolutionary dynamics. Selection will be especially strong if parasites that encounter resistant hosts are destroyed and diluted from the local environment. We tested whether spores of the bacterial parasite *Pasteuria ramosa* were passed through the gut (the route of infection) of their host, *Daphnia magna*, and whether passaged spores remained viable for a “second chance” at infecting a new host. In particular, we tested if this viability (estimated via infectivity) depended on host genotype, whether or not the genotype was susceptible, and on initial parasite dose. Our results show that *Pasteuria* spores generally remain viable after passage through both susceptible and resistant *Daphnia*. Furthermore, these spores remained infectious even after being frozen for several weeks. If parasites can get a second chance at infecting hosts in the wild, selection for infection success in the first instance will be reduced. This could also weaken reciprocal selection on hosts and slow the coevolutionary process.

## Introduction

Coevolution between parasites and their hosts has been implicated in a variety of biological phenomena, including the maintenance of sex (Jaenike 1978; Hamilton 1980; Bell 1982). Host–parasite coevolution requires strong selection on hosts to resist infection and on parasites to infect (Jaenike 1978; Hamilton 1980). Additionally, some degree of genetic matching is assumed to be necessary for successful infection (Clarke 1976; Hamilton 1980; Frank 1993; Agrawal and Lively 2002). For strong selection to result from the interactions, matching parasites must be virulent (Howard and Lively 1994; Lively 2006), and resistant, mismatching hosts should reciprocally impose severe costs on parasites that fail to successfully infect and transmit (Salathé et al. 2008.

In addition to imposing natural selection on parasites, the killing of parasite transmission stages by resistant, unsuitable hosts can lead to a dilution effect, reducing parasite population size and lowering disease prevalence (Ostfeld and Keesing 2000; Schmidt and Ostfeld 2001). This dilution effect has been documented in a *Daphnia-*microparasite system: *Daphnia dentifera* are susceptible to the yeast parasite *Metschnikowia bicuspidata,* whereas the resistant *Daphnia pulicaria* kill the yeast spores in their gut. Consequently, *Metschnikowia* epidemics are smaller in populations where *D. dentifera* coexist with high densities of *D. pulicaria* (Hall et al. 2009).

Are parasites always killed by resistant individuals, or can they get a second chance at infecting a susceptible host? The degree to which resistant individuals kill parasite transmission stages will greatly influence the strength of selection on parasite populations (Salathé et al. 2008), and through dilution effects, the potential for selection on hosts (Keesing et al. 2006). Here, we examined if transmission spores of a sterilizing, bacterial parasite (*Pasteuria ramosa*) can pass through the gut of their freshwater crustacean host (*Daphnia magna*) and remain viable. We used a suite of host genotypes from a population shown to have strong host–parasite genetic specificity (documented by Carius et al. 2001; Luijckx et al. 2011; Auld et al. 2012a). Genetic specificity plays an important role in the infection process of this system. Parasite spores are ingested by potential hosts during feeding, and they do not appear to attach to the esophagus of resistant hosts, but they do attach to the esophagus (presumably the site of infection) of susceptible hosts (Duneau et al. 2011). Subsequently, only the susceptible hosts show a cellular immune response, and this is presumably because it is only in these host–parasite combinations that penetration into the hemocoel occurs (Auld et al. 2012b). We sought to determine if spore viability was reduced during passage through resistant or susceptible *Daphnia* genotypes, and if passaged spores were still viable for a “second chance” in the host population. If these second chance events occur in the wild, there may be significant implications for host–parasite coevolution.

## Material and Methods

### Host–parasite system

*Daphnia magna* (Crustacea: Cladocera) is a planktonic, freshwater crustacean that inhabits freshwater lakes and ponds. It reproduces parthenogenetically, but bouts of sexual reproduction will occur under particular conditions (Kleiven et al. 1992). We maintained *D. magna* under conditions that favored asexual replication, allowing us to establish replicated clonal lines. *Daphnia magna* is the obligate host for *P. ramosa*, a spore-forming bacterial parasite that sterilizes its host and also causes premature death (Ebert et al. 1996). *Pasteuria ramosa* is transmitted horizontally: spores are released from dead hosts and are ingested by *D. magna* during filtration feeding. This study used seven *Daphnia* genotypes and a *P. ramosa* isolate that originated from a pond in Gaarzerfeld, Germany, collected in 1997. This population was used in previous studies showing that infection outcome is dependent on the specific combination of host genotypes and parasite isolates (Carius et al. 2001), and that spores successfully pass from host gut into the hemocoel only in “matching” host and parasite genotype combinations (Auld et al. 2012a). Our experiments were designed to determine (1) if parasites could be passaged live through a host and get a “second chance” to infect another, and (2) if this survival was dependent on the genotype and phenotype of the first host.

### Experimental set-up

Twenty-four replicates of each *D. magna* genotype were maintained for three generations to minimize variation in condition and maternal effects. Five *Daphnia* animals were placed in jars containing 200 mL of artificial medium (Kluttgen et al. 1994) modified using one-twentieth of the SeO_2_ concentration (Ebert et al. 1998) and fed 5.0 ABS (i.e. 1.0 ABS/*Daphnia*) *Chlorella vulgaris* algal cells per day (ABS is the optical absorbance of 650 nm white light by the *Chlorella* culture). Media were changed twice/week. Jars were incubated at 20°C on a 12:12 light:dark cycle. Second/third clutch offspring from the third generation were used in each of two exposure experiments, and individuals were allocated to each treatment at random. *Daphnia* individuals of all genotypes used in a given experiment were the same age.

**Figure 1:**
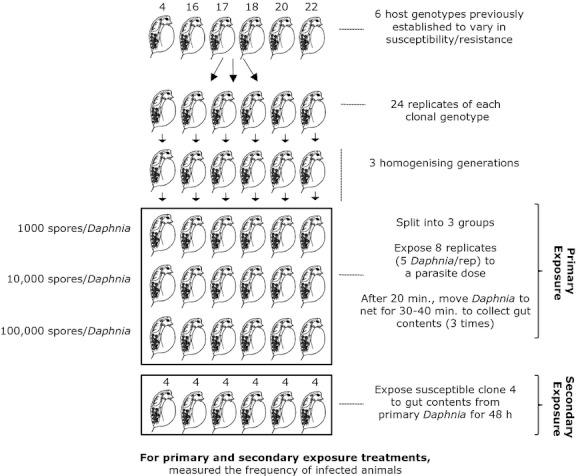
Design of primary and secondary exposure treatments in experiment I. The designs for experiments II and III were similar, but involved different numbers of replicates, primary clonal genotypes, and doses. Additionally, in experiment III, passaged spores from *Daphnia* were frozen for 4 weeks prior to the secondary exposure treatment.

Three experiments examined whether unsuccessful parasites could passage through susceptible and resistant *Daphnia* genotypes and still be capable of initiating new infections (experiments I, II, and III). Each experiment was comprised of two exposure treatments: primary and secondary exposures. In the former, *Daphnia* were exposed to a dose of *Pasteuria* spores, and in the latter, *Daphnia* were exposed to the passaged gut contents of those in the primary exposure treatment. More specifically, in experiment I, six clonal genotypes – two susceptible (clones 4, 17) and four resistant (clones 16, 18, 20, 22) – were exposed to an isolate of *P. ramosa* and then parasite passage was monitored (see experimental design in Fig. 1). Eight replicates of each clone (five *Daphnia* per replicate) were exposed, in a well of a 24-well cell plate (Costar Corning Inc., Corning, NY, USA) to one of three parasite dose treatments: 1000, 10,000, or 100,000 spores/*Daphnia*. In this primary exposure treatment, *Daphnia* were exposed for 20 min. The animals were then moved to a 1.5-mL centrifuge tube placed inside a 100-mL test tube. The centrifuge tube was cut at the bottom and 1-mm mesh placed inside the tube to permit the passaged gut contents of the *Daphnia* to fall through into the test tube, but the animals themselves could not. After 30–40 min, animals were removed from the centrifuge tube and individually returned to the original wells (particles ingested by *D. magna* are defecated after 30–40 min, Evers and Kooijman 1988). *Daphnia* were transferred between the exposure well and collection tubes three times. Before each transfer, the animals were washed thoroughly to remove spores from the carapace. Washing involved placing individual *Daphnia* into clean 500-mL media for approximately 1 min; washing media was refreshed in between each sample.

In the secondary exposure treatment, five new, susceptible individuals (clone 4) per replicate were placed in the 100-mL collection tube with the primary *Daphnia* passaged gut contents for 48 h. Primary and secondary *Daphnia* were placed in jars with 200-mL fresh media and maintained for 30 days post-exposure whereupon they were assessed for evidence of infection. Infection is easily determined by eye because *Daphnia* have a transparent carapace: *Pasteuria-*infected hosts have an empty brood chamber and their hemolymph is an opaque red/brown, whereas uninfected hosts have either developed ovaries or a brood chamber containing offspring and their hemolymph is clear. Animals in the primary exposure treatment were assessed for infection to confirm that the parasite isolate used was able to infect (previously-determined) “susceptible” genotypes, but not “resistant” genotypes.

To verify that *Daphnia* clones were indeed passing viable spores, the experiment was repeated, but this time at a higher dose with an expanded number of primary clonal genotypes. In experiment II, 12 replicates of three susceptible clones (clones 4, 17 from experiment I, and clone 3) and three resistant clones (clones 16, 18 from experiment I, and clone 23) were exposed to 1 million spores/*Daphnia* during the primary exposure treatment. Susceptible clone 4 was used in the secondary exposure treatment as described above. As in experiment I, all *Daphnia* were placed in jars with 200 mL fresh media post-exposure, maintained for 30 days, and then assessed for infection.

In experiment III, we examined whether the passaged spores were viable over time under more extreme external conditions. In the primary exposure treatment, 12 replicates of susceptible clone 4 and resistant clone 16 (five *Daphnia*/rep.) were exposed to parasite spores at two doses: 100,000 and 1 million spores/*Daphnia*. Secondary exposures were performed similarly as above, except that there was a time delay between the primary and secondary exposures. We placed the passaged gut contents in a freezer at −20°C for 4 weeks. In this way, *Pasteuria* spores (if present) were frozen quickly to a lower temperature, and for a longer duration, than they would normally experience in their own pond. The use of a more extreme condition allowed us to robustly test whether being passaged affected the viability of spores, regardless of environmental condition. Following this, clone 4 animals (this time with each replicate having only one *Daphnia* host) were exposed for 48 h to the thawed gut contents collected from the primary exposure treatment, and the animals were observed for infection at 30 days.

### Statistical analyses

Data were analyzed using IBM SPSS Statistics 20.0 (Armonk, NY) using generalized linear models, with identity link function. The response variable was the proportion infected in each jar, and this was arcsine-square-root transformed. For experiment I, we first tested if primary clonal phenotype (susceptible/resistant), genotype (nested within phenotype), dose, and interactions with dose determined the proportion that became infected during the primary and secondary exposure treatments. At no time did we compare infection levels in primary and secondary exposures. The analysis for experiment II mirrored the above analysis for I, but no dose effects were studied. For the primary exposure treatment in experiment III, a generalized linear model was used with phenotype and dose as fixed factors.

## Results

**Figure 2:**
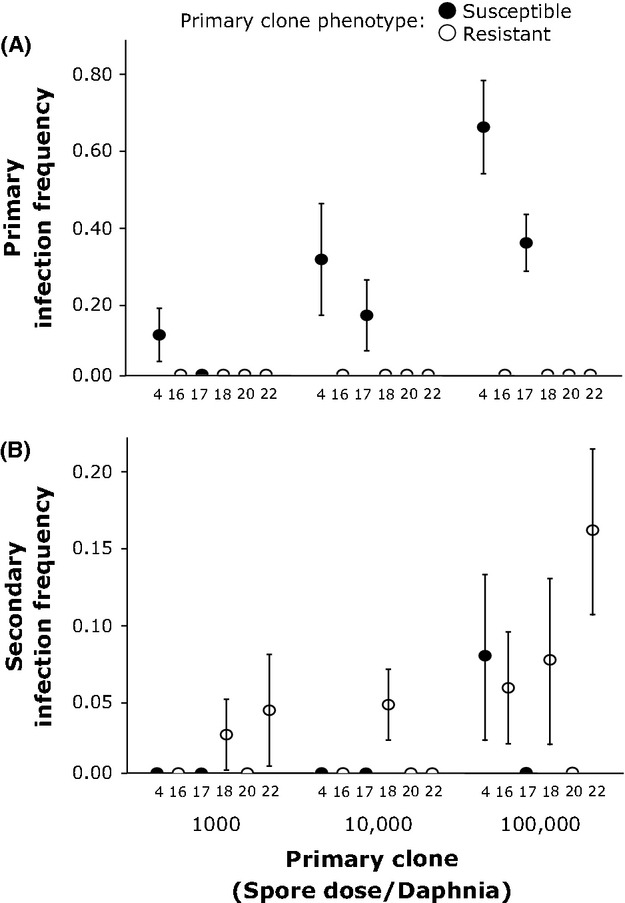
Infection frequencies in (A) primary and (B) secondary exposure treatments in experiment I. Three parasites doses were used: 1000, 10,000, and 100,000 spores/*Daphnia*. Infection frequencies are presented for six clonal genotypes (4, 16, 17, 18, 20, 22) in the primary exposure treatment and the clones (all 4) exposed in the secondary exposure treatment. The phenotypes (susceptible, resistant) of primary clones are shown. Error bars represent ± 1 SE. Note the *y*-axis scales are different between graphs.

**Figure 3:**
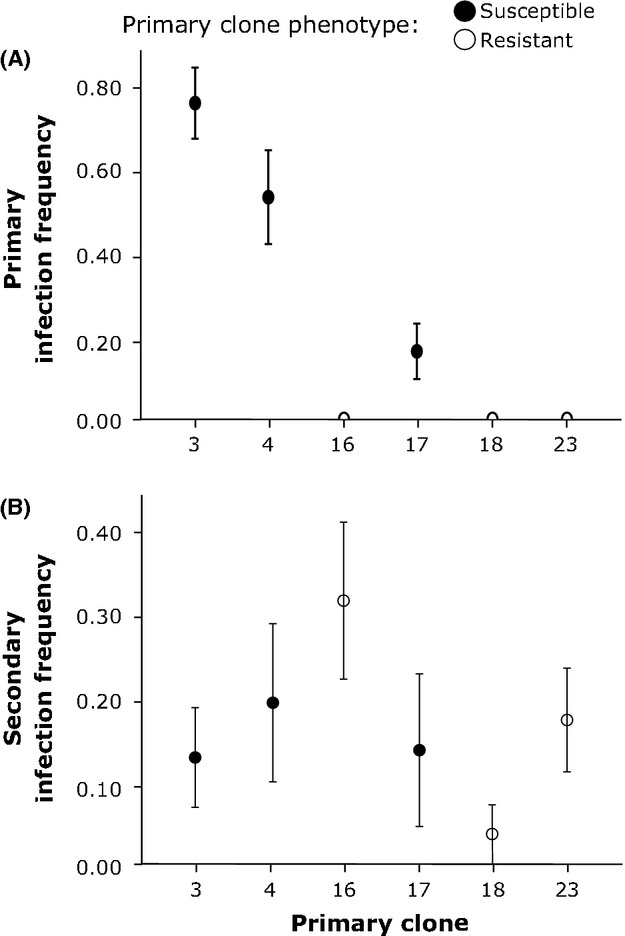
Infection frequencies in (A) primary and (B) secondary exposure treatments in experiment II. One parasite dose was used: 1 million spores/*Daphnia*. Infection frequencies are presented for six clonal genotypes (3, 4, 16, 17, 18, 23) in the primary exposure treatment and the clones (all 4) exposed in the secondary exposure treatment. The phenotypes (susceptible, resistant) of primary clones are shown. Error bars represent ± 1 SE. Note that the *y*-axis scales are different between graphs.

Across the primary exposure treatments in experiments I, II, and III, infection patterns were as expected: clones previously known to be susceptible became infected, while resistant clones remained uninfected (Tables 1–3; Figs. 2A, 3A). Infection frequencies of susceptible clones increased with dose, and ranged from 0% to 15%, on average, at the lowest dose in experiment I to 20–78% at the highest dose in experiment II. In addition, for the primary exposure treatment, dose, clonal genotype (nested within phenotype), and the interaction between phenotype and dose were significant in experiment I (Table 1). Clonal genotype was a significant factor in experiment II (Table 2).

**Table 1:** Generalized linear model for effects of primary clonal phenotype, genotype, and dose on infection frequency in primary and secondary exposures in experiment I

Source	df	Wald χ^2^	*P*
Primary exposure			
Intercept	1	99.341	<0.001
Phenotype	1	113.911	<0.001
Clone (Phenotype)	4	16.953	0.002
Dose	2	50.744	<0.001
Phenotype × Dose	2	58.221	<0.001
Clone (Phenotype) × Dose	8	3.154	0.924
Secondary exposure			
Intercept	1	16.821	<0.001
Phenotype	1	3.456	0.063
Clone (Phenotype)	4	14.965	0.005
Dose	2	13.987	0.001
Phenotype × Dose	2	0.640	0.726
Clone (Phenotype) × Dose	8	16.840	0.032

**Table 2:** Generalized linear model for effects of clonal phenotype and genotype on infection frequency in primary and secondary exposures in experiment II

Source	df	Wald χ^2^	*P*
Primary exposure			
Intercept	1	128.474	<0.001
Phenotype	1	128.474	<0.001
Clone (Phenotype)	4	46.993	<0.001
Secondary exposure			
Intercept	1	34.761	<0.001
Phenotype	1	0.461	0.497
Clone (Phenotype)	4	9.123	0.058

**Table 3:** Effects of clonal phenotype and dose on infection frequency in primary and secondary exposures in experiment III

Source	df	Wald χ^2^	*P*
Primary exposure			
Intercept	1	445.052	<0.001
Phenotype	1	445.052	<0.001
Dose	1	0.027	0.868
Phenotype × Dose	1	0.027	0.868

	100,000 spores/*Daphnia*		1 million spores/*Daphnia*	
		
Secondary exposure	Susceptible primary	Resistant primary	Susceptible primary	Resistant primary
Infected replicates (out of 12 total)	8	8	8	9

Infections in the secondary exposures of all three experiments were not significantly affected by the primary clonal phenotype (Tables 1–3). In experiment I, secondary infections ranged from 0% to 32% and were dependent on dose (Table 1 and Fig. 2B). The genotype of the primary clone also affected the likelihood of the secondary clone becoming infected (Table 1), except in experiment II (Table 2). The number of clonal genotypes releasing parasites increased with dose (Fig. 2B), and all clones, regardless of genotype, released parasites at the highest dose (Fig. 3B).

Passaged parasites remained viable after being frozen for several weeks. In the primary exposure treatment of experiment III, resistant clone 16 was uninfected at both doses, and susceptible clone 4 was infected, on average, 90% and 86% at doses of 100,000 spores/*Daphnia* and 1 million spores/*Daphnia*, respectively. There was little difference in the number of infected *Daphnia* in the secondary exposure treatment, across replicates (Table 3).

## Discussion

Strong host-mediated selection on parasite populations is an important component of coevolution (Hamilton 1980; Lively 1999; Salathé et al. 2008). Hosts can pay substantial fitness costs of infection, but less is known of the impact of resistant individuals to parasite fitness (except see King et al. 2011). Parasites may fail to infect hosts for two reasons: killed by host defenses (e.g., by a successful host immune response) or repelled by non-lethal host barrier defenses (Parker et al. 2011). Host–parasite interactions where resistant individuals kill parasites will generate stronger selection than those where unsuccessful parasites regularly have a “second chance” at infecting. Our results for the *Daphnia–Pasteuria* system show that many parasite transmission stages can pass through hosts and remain qualitatively infectious, even after being frozen for several weeks.

The degree of secondary infection did not depend on whether the first host was resistant or susceptible to *P. ramosa* infection (Figs. 2B, 3B). In this system, spores are activated once ingested, and those that match “attach” to the host esophagus and infection ensues in susceptible but not in resistant combinations (Duneau et al. 2011; Auld et al. 2012a). This matching should reduce the number of spores that could be passed through the gut, and it was somewhat unexpected that both susceptible and resistant host genotypes released viable parasites. Spores were rarely released, viable or otherwise, at lower parasite doses, but at higher doses, all host genotypes released spores (Fig. 3B). Potentially, too many matching parasites trying to attach at high doses overload the attachment mechanism and cause inactivated spores to pass through susceptible hosts. After a failure to attach, digestion may kill some spores (this number is difficult to quantify), but our experiments at least suggest that many viable spores are still passed. Infection in the secondary exposure was, however, influenced by host genotype in the first. Genetic variation for host capacity to kill parasite spores requires more exploration, given the implications for selection. A number of mechanisms could underlie this genetic variation; for example, host genotypes may simply differ in their feeding or excretion rates, or immune system efficiency.

Millions of *Pasteuria* spores can be produced by one infected *D. magna* individual (Ebert et al. 2000; Carius et al. 2001; Vale and Little 2009), and as epidemics intensify in a pond habitat, billions of *Pasteuria* spores could be in the aquatic environment. Thus, we think it is likely that second chances may be common in some *Daphnia* populations. Other parasite species are, however, unlikely to have a second chance at infection. Spores of the yeast parasite *M. bicuspidata* are killed when ingested by the wrong *Daphnia* species (Hall et al. 2009), and spores of the microsporidian parasite *Glugoides intestinalis* are killed when ingested by the wrong *D. magna* genotype (Pulkkinen 2007). With experimental procedures comparable to the current study, King et al. (2011) found that trematode parasites either infected susceptible snails or were killed by resistant snails of the same species in sympatric and allopatric populations.

The fact that *P. ramosa* spores are not always killed by resistant hosts may have consequences for the strength of selection in this system, which is renowned for its coevolutionary dynamics (Decaestecker et al. 2007). The weakened selection resulting from parasites having multiple chances at infection will generate a knock-on effect for selection on hosts because presently unsuccessful parasite genotypes are not always removed from the population. Moreover, the ability of parasites to safely passage through resistant hosts may be linked to the effects of parasite longevity. The ability of a parasite to persist in the external environment provides it with a better opportunity to “sit and wait,” and under some circumstances, this strategy will favor higher virulence (Bonhoeffer et al. 1996; Gandon 1998; Roche et al. 2011). Generally, the consequences of a “second chance” for parasites, as well as for hosts, may vary both within and across host–parasite systems, but are worth examining further given the potential for impact on the coevolutionary process.
